# The Effect of Lens Shape, Zonular Insertion and Finite Element Model on Simulated Shape Change of the Eye Lens

**DOI:** 10.1007/s10439-024-03491-3

**Published:** 2024-03-19

**Authors:** Lin Ye, Kehao Wang, Jorge Grasa, Barbara K. Pierscionek

**Affiliations:** 1 Faculty of Health Education Medicine and Social Care, Medical Technology Research Centre, Anglia Ruskin University, Chelmsford Campus, Chelmsford, UK; 2 Beijing Advanced Innovation Centre for Biomedical Engineering, Key Laboratory for Biomechanics and Mechanobiology of Ministry of Education, School of Engineering Medicine, Beihang University, Beijing, China; 3 Aragon Institute of Engineering Research (i3A), University of Zaragoza, Zaragoza, Spain; 4 Bioengineering, Biomaterials and Nanomedicine Networking Biomedical Research Centre (CIBER-BBN), Zaragoza, Spain

**Keywords:** Eye lens, Finite element modelling, Zonular insertion, Refractive index

## Abstract

The process of lens shape change in the eye to alter focussing (accommodation) is still not fully understood. Modelling approaches have been used to complement experimental findings in order to determine how constituents in the accommodative process influence the shape change of the lens. An unexplored factor in modelling is the role of the modelling software on the results of simulated shape change. Finite element models were constructed in both Abaqus and Ansys software using biological parameters from measurements of shape and refractive index of two 35-year-old lenses. The effect of zonular insertion on simulated shape change was tested on both 35-year-old lens models and with both types of software. Comparative analysis of shape change, optical power, and stress distributions showed that lens shape and zonular insertion positions affect the results of simulated shape change and that Abaqus and Ansys show differences in their respective models. The effect of the software package used needs to be taken into account when constructing finite element models and deriving conclusions.

**Supplementary Information:**

The online version contains supplementary material available at 10.1007/s10439-024-03491-3.

## Introduction

The human eye lens has been studied from biological, optical and more recently biomechanical aspects to try and understand structure and function relationships that can explain the process of accommodation: how the lens changes shape to adjust the focussing power of the eye and its gradual loss with age, presbyopia. The classical explanation of Helmholtz [1] has been challenged and most recently so by the theory of Schachar [2] for which supporting evidence has been found [3, 4]. As the ciliary muscle contracts and relaxes, it alters the tension on the zonular fibres attached to the lens capsule thereby altering the shape and thickness of the lens allowing it to change the focus of the eye to meet visual demands. The fundamental differences in the theories of Helmholtz and Schachar are in the contribution of the various sections of the zonule to the change in shape of the lens. The changes in zonular tension as the lens alters shape are very difficult to measure in the living eye. Hence, finite element modelling has been applied to complement experimental studies and glean more about the mechanism of accommodation. It is vital that models are constructed based on biological data and biometric parameters of the lens as well as accommodative components in order to represent the physiologically situation.

However, no models to date can claim to be truly representative of the accommodative system let alone to understand individual variations in the component structures that mediate and control the forces of accommodation. The zonule has been modelled as an element with two or three branches that meeting at the same point [5–9]. Biologically, the zonule comprises many ligaments in separate bundles and these ligaments are joined or inserted at one end to the capsule of the lens and at the other to the ciliary muscle. The positions of insertion are not known and could vary between individuals. Recent modelling work has indicated that the insertion positions of the zonule have a significant impact on the forces mediated to the lens and on the consequent shape changes induced [3, 10, 11].

Modelling requires accurate material properties, and these have largely come from the seminal work of Fisher [12] who measured material properties of different aged lenses using centrifugal force to alter the shape of the lens in order to simulate accommodation. Fisher reported that the elastic modulus of the lens nucleus was smaller than that of the cortex [12]. More recently, Wilde et al. [13] used similar experimental methods and found that in younger lenses, the nuclear shear modulus was smaller than that of the cortex, indicating that the cortex was stiffer at this stage, but that from about age 45 onwards, the nucleus was stiffer than the cortex [13]. Brillouin scattering analysis has also been used to measure longitudinal modulus of the lens in living eyes and this was found to correlate highly with the profile of refractive index in the lens [14].

Variations in results, with regard to material properties, have been reviewed and shown to be dependent on experimental methods used [10]. Yet to date, there have not been any studies that have compared models created by different software to elucidate whether variations in software principles and algorithms can have a discernible effect on results of modelling lens shape change. Any differences in modelling arising from software used need to be known so that there is no over reliance on any single software model type in trying to improve understanding of accommodation, presbyopia and/or for aiding design of intraocular implants.

## Methods

Three-dimensional quarter lens geometric models were developed using 3D CAD software SolidWorks (ver. 2021), which were then imported into Finite Element Analysis software Abaqus (ver. 2022) and Ansys workbench (ver. 2021R2) for discretization and FE model development.

**Fig. 1:**
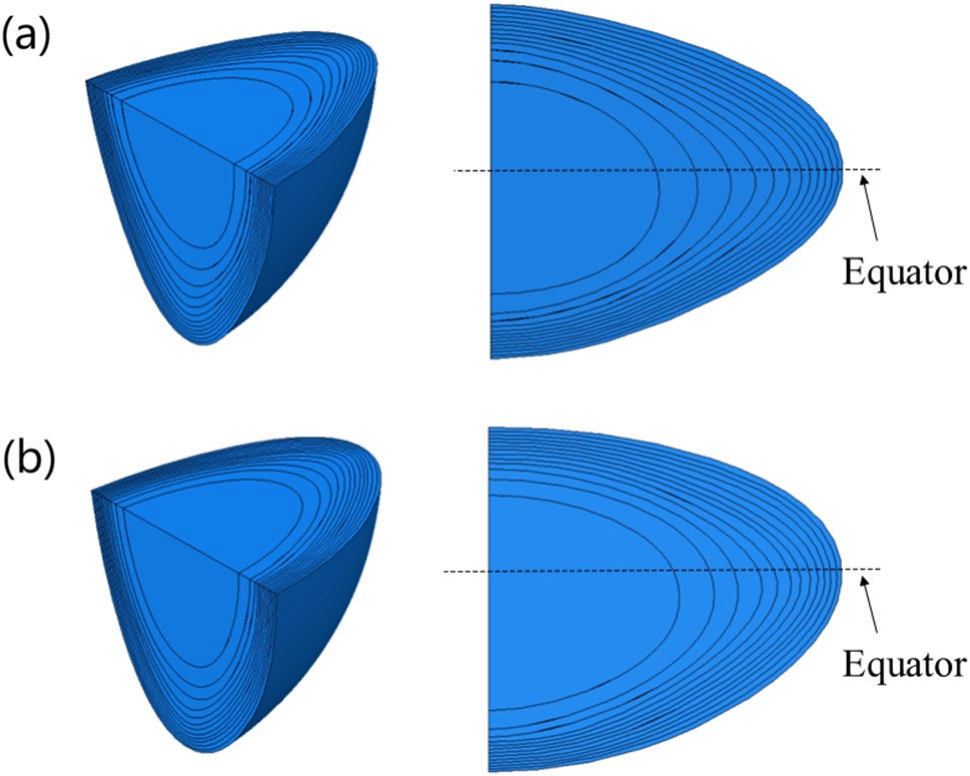
Finite element models of two 35-year-old lenses, in which **a** is symmetric and **b** is asymmetric.

The geometries of developed models were based on the images obtained from measurements of human lens refractive index contour profiles [15]. Two 35-year-old lenses with different shapes were selected to construct 3D models (Fig. 1). The purpose of using two lenses from the same age with different shapes is twofold: it indicates that age is not a determining factor in lens biometry and physiology, and it allows investigation of how such differences may affect results of models. In one of the lenses, the anterior and posterior curvatures are similar resulting in the equatorial plane being approximately central (the symmetrical lens) (Fig. 1a); the other has a posterior surface that is more curved than the anterior resulting in the equatorial plane being shifted anteriorly (the asymmetrical lens) (Fig. 1b).

## Geometry of the Model

**Fig. 2:**
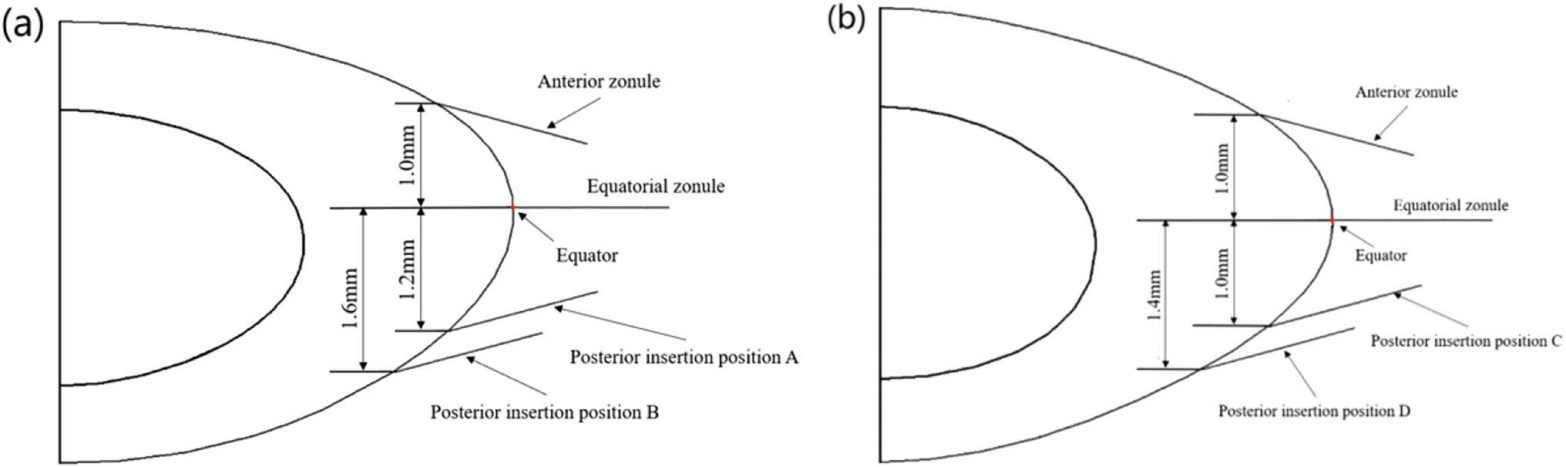
Zonular insertions showing the two variations in posterior zonular position in the **a** asymmetric model and **b** symmetric model.

**Fig. 3:**
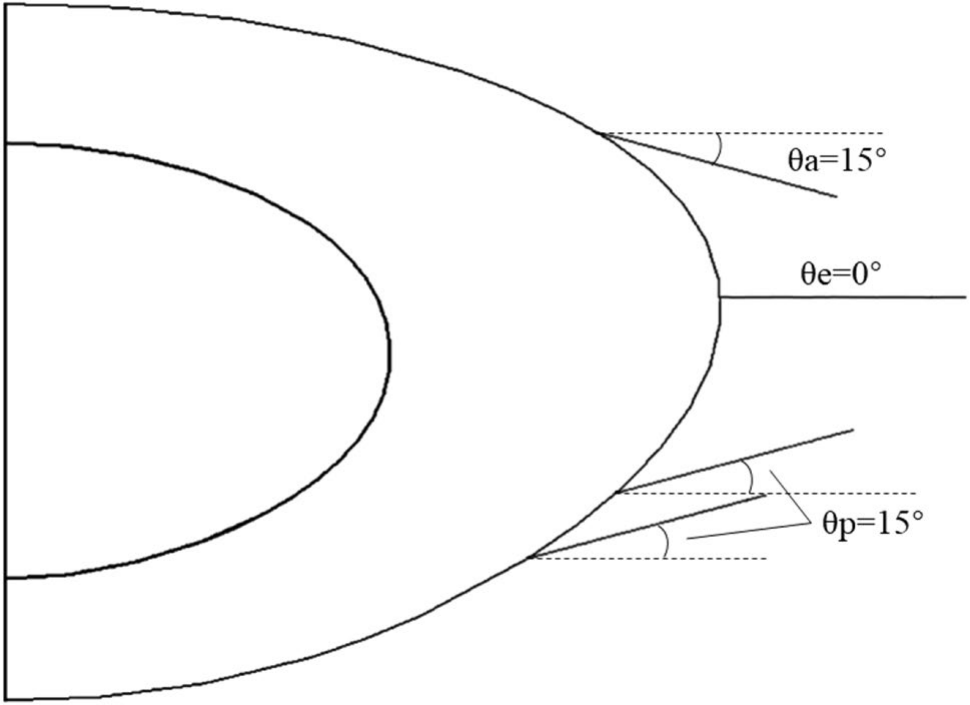
The insertion angles of zonular fibres (sagittal view)

Each model consists of the lens nucleus, the lens capsule, zonular fibres and the lens cortical sections which were divided into 12 layers (Fig. 1), according to the respective refractive index profiles [15]. The zonule was separated into three sections: the anterior, the equatorial and the posterior zonular sections. Each section consists of 17 cylinders, representing zonular fibres, each of which was 1.5 mm in length and 0.025 mm in radius [3]. The capsule in each model has a constant thickness of 6 microns [16]. The zonular fibres are attached to the capsule in the equatorial plane i.e. 0° to the lens, and at an angle of 15 ° to the equator in anterior and posterior directions (Figs. 2 and 3).

In order to test how zonular anchorage position influences shape change, optics and biomechanics, two different locations of insertion points were selected for each lens model. For both models, the point on the lens contour with a tangent slope of 0° was chosen as the insertion point of the equatorial zonule. The insertion position of the anterior zonule was obtained according to the formula provided by Sakabe et al. [17], and the insertion position of the posterior zonule was selected within the range of its insertion position [18]. For the asymmetric model, the insertion point of the anterior zonule was selected at a distance of 1.0 mm from the equatorial plane. The insertion points of the posterior zonule were selected at locations 1.2 mm and 1.6 mm from the equatorial plane. For the symmetric model, the insertion points of anterior zonule were at a distance of 1.0 mm from the equatorial plane and locations of 1.0 mm and 1.4 mm from the equatorial plane were selected as different insertion positions of the posterior zonule. The insertion positions of the two posterior zonule in the asymmetric model were named position “A” and “B”, respectively, and the insertion positions of the two posterior zonule in the symmetric model were named “C” and “D”, respectively. The same geometric parameters were used to build models in both Abaqus and Ansys.

## Material Properties

Each layer of each model was assigned a different Young's modulus based on the data from Fisher for 35-year-old lenses [12] and distributed in accordance with the gradient index profiles previously measured for these lenses [15]. This was done by adding 0.5 MPa per layer from the innermost to the outermost layer to make the average for all layers concur with the equivalent modulus from Fisher's data (Table 1). Young's modulus was 0.35 MPa and 1.5 MPa for the zonule and capsule, respectively [19, 20]. Poisson's ratio was 0.49 for the entire lens and 0.47 for the capsule and zonule [12, 21].

**Table 1:** Young's modulus of each layer of the lens according to Fisher's data

	Young's modulus for each layer of lens models (KPa)							Average modulus of cortex (Kpa)
	Nucleus	Cortex						
Layer	1	2	3	…	11	12	13	
Modulus	0.6	1.1	1.6	…	5.6	6.1	6.6	3.85

## Mesh and Boundary Conditions

The lens was meshed using 8-node solid element (ANSYS element type: Solid185, KEYOPT(3) = 0, KEYOPT(6) = 1; ABAQUS element type: C3D8H). The capsule was discretized using 4-node membrane element (ANSYS element type: Shell181, KEYOPT(3) = 0; ABAQUS element type: M3D4H). In both Abaqus and Ansys, the capsule was set as a skin (or face-coating) during meshing, so the shape of the capsule mesh followed the lens mesh. The zonule was modelled as 2-node beam element (Ansys element type: Beam188, KEYOPT(1) = 0, KEYOPT(3) = 0, Abaqus element type: B31H).

The asymmetric Ansys model contains 70703 nodes and 68787 elements; the asymmetric Abaqus model contains 73127 nodes and 71187 elements. The symmetric Ansys model contains 70670 nodes and 68787 elements; the symmetric Abaqus model contains 71708 nodes and 69811 elements. A mesh independence analysis was performed for both software types.

The two orthogonal cross-sectional planes of each lens model were set as symmetrical planes, so that the quarter model had the same deformation effect as the full model. The centre of the lens was constrained in all degrees of freedom. The free end (node) of each zonule was set to move in a radial direction, whilst the end (node) in contact with the lens was connected to the capsule on the lens surface. In Abaqus, a coupling constraint was used to connect the node of zonule with the surrounding capsular nodes. In Ansys, a bond contact relationship was used, and Multiple Points Constraint (MPC) formulation was selected to connect the zonular node to the same capsule nodes as the nodes selected in Abaqus. Kinematic coupling in Abaqus constrains the motion of the coupling nodes to the rigid body motion of the reference node and eliminates degrees of freedom at the coupling nodes. The MPC formulation in Ansys adds a connection between nodes to limit the degree of freedom of the nodes. There is no major difference in principle between the two methods of contact.

A displacement of 0.5 mm was applied to each zonule and the displacement on each zonule was in the radial direction. Stresses are shown as von Mises stresses.

## Calculation of Optical Power

For each combination of zonular insertion points, the lens surface shape was extracted and nodes within the central region of 6 mm diameter were fitted using the curve fitting tool in MATLAB (ver.2019).

The Central Optical Power (COP) of the lens models was calculated based on Eq. (1)

1 $$COP = \frac{{n_{1} - n_{\text{a}} }}{{r_{\text{a}} }} + \frac{{n_{1} - n_{\text{a}} }}{{r_{\text{p}} }} - \frac{{t\left( {n_{1} - n_{\text{a}} } \right)^{2} }}{{r_{\text{a}} r_{\text{p}} n_{1} }},$$

where *n*_a_ = 1.336 is the refractive index of aqueous humour, *n*_1_ = 1.42 is the equivalent refractive index of the lens [9, 22], *r*_a_ and *r*_p_ are the anterior and posterior radius of curvature and t is the thickness of the lens model.

The radii of curvature of the anterior and posterior surfaces were obtained by fitting the circle using the least square method for the axial and paraxial regions (extending 1.5 mm either side of the optic axis) in MATLAB (ver.2019). The thickness of the lens was obtained by calculating the length of the central axis of the model.

## Results

**Fig. 4:**
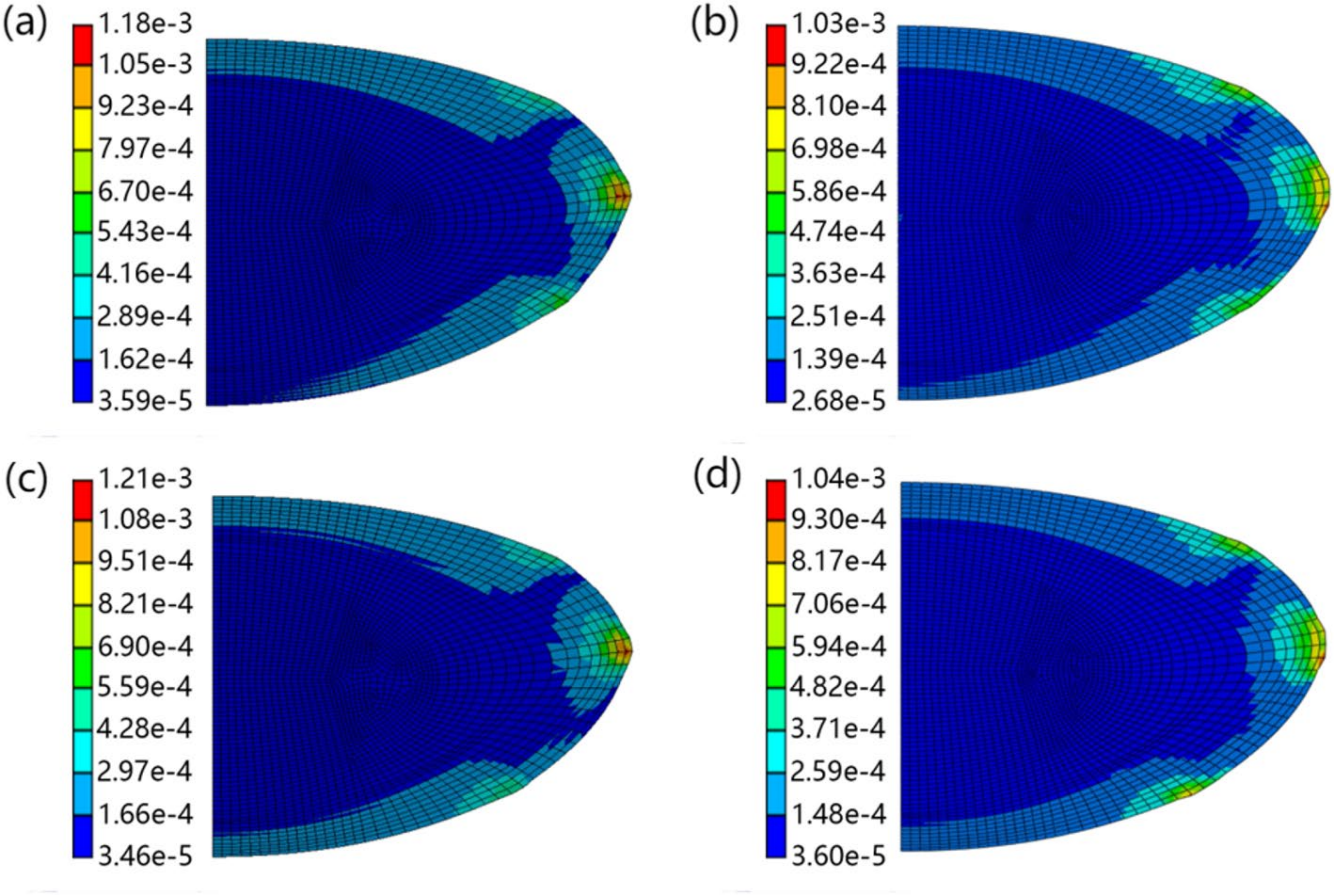
Stress distribution results of the asymmetric lens models showing two zonular combinations for the models in Ansys and Abaqus, where **a** shows the model using combination A, calculated in Ansys, **b** shows the model using combination A, calculated in Abaqus, **c** shows the model using combination B, calculated in Ansys, **d** shows the model using combination B, calculated in Abaqus

**Fig. 5:**
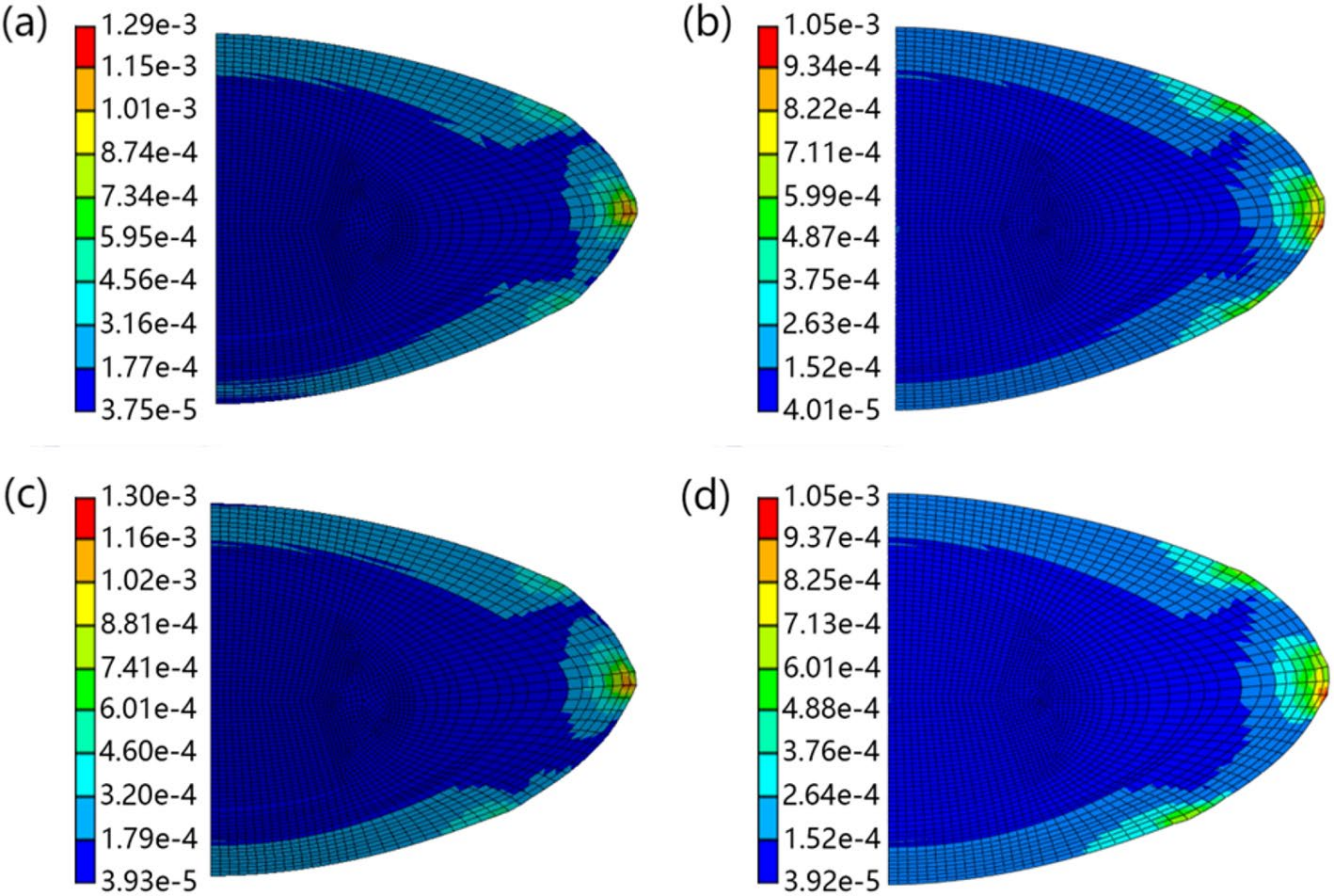
Stress distribution results of the symmetric lens models each used two zonular combinations in the two software. where **a** shows the model using combination C, calculated in Ansys, **b** shows the model using combination C, calculated in Abaqus, **c** shows the model using combination D, calculated in Ansys, **d** shows the model using combination D, calculated in Abaqus.

The stress distributions of the asymmetric and symmetric lens models after simulated stretching, with two zonular combinations, in Ansys and Abaqus software are shown in Figs. 4 and 5, respectively. Different colours represent different stress ranges, with specific von Mises stress values in Megapascals (MPa) shown. The stress distributions vary between models constructed in Ansys and in Abaqus. For the asymmetric lens (Fig. 4), the maximum stress in the Ansys model is greater than the maximum stress in the Abaqus model for both zonular combination A (1.18e-4 MPa compared to 1.03e-3 MPa in the Abaqus model) and combination B (1.21e-3 MPa compared to 1.04e-3 MPa in the Abaqus model). The high stress of the Ansys model is mainly reflected around the equatorial region (Fig. 4a and c). There is a more defined stress variation in the cortical region around the equator in models constructed with Ansys for both zonular combinations (Fig. 4a and c) compared with their counterparts in Abaqus in which the stresses are more distributed (Fig. 4b and d). In the two zonule combinations, stretching of the zonule in Abaqus models produced a larger range of stress increases (Fig. 4b and d). The effect of varying posterior zonular insertion points is seen when comparing Fig. 4a with c or b with d. With the insertion position of the posterior zonule placed further from the equator (Fig. 4c and d), the stresses are more distributed than for models in which the posterior zonule is closer to the equator (Fig. 4a and b).

The contours of stress are less widely distributed in the symmetric (Fig. 5) than the asymmetric models (Fig. 4), especially around the anterior and posterior pole. The maximum stress magnitudes are higher in models constructed with Ansys than the same models constructed in Abaqus (seen in comparison of Fig. 5a with c and Fig. 5b with d). There are several localized stress concentrations around the cortico-nuclear region in the models constructed with Ansys (Fig. 5a and c); these are not seen in the models constructed with Abaqus (Fig. 5b and d). In Ansys models (Fig. 5a and c), lower stress region can be seen between zonular insertion positions, which is not obvious in Abaqus models (Fig. 5b and d). The zonular stretching appears to have a greater effect on the localized stress distribution in Abaqus models than in Ansys models.

The radii of curvature of the anterior and posterior surfaces and the calculated Central Optical Power (COP) for both lenses before and after simulated stretching are shown in Table 2. The results show that because the symmetric lens models have more curved surfaces and hence larger initial COP values than the asymmetric lens models. For both models in Ansys and Abaqus, the COP of the symmetric model changed more than the asymmetric model after simulated stretching.

**Table 2:** Radii of curvature of anterior and posterior lens surfaces and Central Optical Power (COP) of models

Model and software	Zonular insertion combination	Surface	Initial radius (mm)	Radius after stretching (mm)	Initial COP (D)	COP after stretching (D)	Variation of COP (D)
Ansys asymmetric model	A	Anterior	10.96	13.16	19.48	16.53	2.95
		Posterior	6.94	8.13			
	B	Anterior	10.96	13.35		15.63	3.85
		Posterior	6.94	8.84			
Ansys symmetric model	C	Anterior	5.33	6.99	31.75	25.09	6.66
		Posterior	5.00	6.21			
	D	Anterior	5.33	7.00		24.11	7.64
		Posterior	5.00	6.71			
Abaqus asymmetricmodel	A	Anterior	10.96	13.03	19.48	16.74	2.74
		Posterior	6.94	8.01			
	B	Anterior	10.96	13.23		15.8	3.68
		Posterior	6.94	8.73			
Abaqus symmetric model	C	Anterior	5.33	6.89	31.75	25.47	6.28
		Posterior	5.00	6.12			
	D	Anterior	5.33	6.89		24.48	7.27
		Posterior	5.00	6.61			

Comparing the different zonular combinations, there is a greater change in COP with stretching when the posterior zonule is further from the equator for both lenses and both software models. There is an increase in 0.9D to 1D in COP when the posterior zonule moves further from the equator in all models (Table 2). When results of simulated stretching in Abaqus and Ansys are compared there is very little difference in change of COP for simulated stretching in Ansys than in Abaqus for both lenses and both zonular combinations. The differences in COP between Ansys and Abaqus are 0.21D and 0.17D for zonular combination A and B, respectively, in the asymmetric lens, and 0.38D and 0.37D for zonular combination C and D, respectively, in the symmetric lens.

The changes in lens shape with simulated stretching for zonular combinations A and B applied to the asymmetric model in Ansys and Abaqus software are shown in Supplementary Figs. S1 and S2. The displacement of anterior pole, posterior pole, and equator is shown in Table 3.

**Table 3:** Anterior and posterior shift in different asymmetric models

Model	Ansys		Abaqus	
Combination	A	B	A	B
Anterior shift (mm)	0.1403	0.1446	0.1316	0.1391
Posterior shift (mm)	0.1769	0.1909	0.1661	0.1796
Equatorial shift(mm)	0.2033	0.1981	0.1751	0.1692

The shifts of the poles and hence the deformation of the lens with simulated stretching are greater for zonular combination B than for A i.e. when the posterior zonule is further away from the equator. The deformation of the posterior surface is larger than that of the anterior surface, and greater for models in Ansys than in Abaqus (Supplementary Figs. S1–S4). As the posterior zonule moves away from the equator, the displacement of both the anterior and posterior poles increases, with the latter showing the greater displacement. The equatorial displacement decreases as the posterior zonule moves away from the equator in the Ansys and Abaqus models, with the latter showing the smaller displacement.

Supplemental Fig. S3 and S4 show the changes in shape of the symmetrical lens with simulated stretching for both zonular combinations C and D with Ansys and Abaqus. The displacement of anterior pole, posterior pole and equator is shown in Table 4. The anterior and posterior poles of the symmetric model deform more under the same displacement than the asymmetric model for both Ansys and Abaqus. Conversely, the equatorial displacement of the asymmetric models is slightly greater than that of the symmetric models.

**Table 4:** Anterior and posterior shift in different symmetric models

Model	Ansys		Abaqus	
Combination	C	D	C	D
Anterior shift (mm)	0.1776	0.1819	0.1668	0.1721
Posterior shift (mm)	0.1911	0.2059	0.1799	0.1954
Equatorial shift(mm)	0.2007	0.1946	0.1737	0.1672

## Discussion

In this study, several models based on the refractive index profiles of two human lenses of the same age obtained by X-ray phase contrast tomography were constructed using two finite element analysis software packages. The results show the influence of lens shape and zonular insertion positions on lens optical power and on stress distributions with simulated shape change of the lens.

When comparing stress distributions after simulated shape change, for both asymmetric (Fig. 4) and symmetric lens models (Fig. 5), the stress distributions were similar in Ansys and in Abaqus. However, the stress ranges vary between software models for both lenses; the main difference in stress can be found in the zonular insertion region, especially around the equator (Figs. 4 and 5). The difference in deformation of the lens at the zonular insertion position is seen in Supplementary figures, where combination A of Supplementary Fig. S1 and combination C of Supplementary Fig. S3 show a more obvious difference in the calculation results of the two software types.

Stress is a measure of internal resistance of a material in response to external forces and in the case of the eye lens, the distribution of stress is determined by a range of factors including the lens geometry, internal stiffness distribution, the angle of zonular force and location of zonular anchorage position on the lens capsule and ciliary muscle [10]. The influence of material properties on the distribution of stress has been demonstrated previously using models developed both in Ansys [22] and in Abaqus [4]. Models developed with a uniform stiffness across the whole lens tend to develop a stress concentration region near the nuclear-cortical boundary [4, 22]; such high stresses can be gradually reduced with an increasing number of cortical layers of gradient elastic moduli [22] and be eventually eliminated with models of linearly changing cortical elastic moduli [3]. Indeed, models in which stresses are well distributed would be of great advantage in further studies of lenses with irregularities in structure such as those resulting from opacifications seen with cataract. In such cases, localised stresses that represent pathological features could be properly distinguished. Stress distributions demonstrated by models developed in the present study correspond well with those reported previously apart from the high stress region in the lens equator (Figs. 4 and 5). This may be attributed to the different lens shapes and different constraint methods adopted in the present study.

The developed models have demonstrated differences in curvature and COP change with simulated stretching between Ansys and Abaqus models with COP change of up to 7.64D, which corresponds to the accommodative range of lenses from the fourth decade [23]. When comparing the shape changes caused by simulated stretching, the results showed that for both the asymmetric (Supplementary Figs. S1 and S2) and symmetric models (Supplementary Figs. S3 and S4), a greater amount of shape change was produced for models created in Ansys than for models created in Abaqus. The models for the symmetric lens produced greater changes in COP after simulated stretching than their counterpart asymmetric lens models (Table 2) and this concurs with findings from a previous study which showed that lens geometry changes play an important role during accommodative loss [24]. The lens continues to grow with age by continued addition of lens fibre cells, but these are not necessarily equally elongated across the anterior and posterior surfaces, hence the manifestation of this growth process is that the older lenses can be more asymmetric than younger lenses [15]. In addition, individual variations exist [15] as seen in the two lenses from which models were derived in this work. For both asymmetric and symmetric lenses, models created in Ansys and Abaqus showed greater shape and COP changes for zonular combinations where the posterior zonular insertion is further from the equator (combinations B and D) than when it is closer to the equator (combinations A and C). These differences in COP are at the level of clinical significance: around 0.9D in asymmetric model and 1D in symmetric model (Table 2).

Wang at al [3] have shown that the different zonular angles can have a large impact on the results. This study confirms these previous findings and shows additionally that lens shape, which is not necessarily indicative of lens age, has a significant effect on results of modelling simulated shape change. This has implications for modelled distribution of stresses and for change in optical power. These findings collectively indicate that models created in Ansys manifest greater deformation and local stresses around the points of zonular insertions and stresses that are not as well transferred across the lens surface than for models generated in Abaqus, even with the use of constraints and mesh that are as similar as possible. Well-distributed stresses over the lens surface may confer a biological advantage because this would reduce localised areas of high stress that could adversely affect tissue function. Whilst the differences in models created by the two software types are linked to intrinsic settings within the packages, it is vital to understand what effect such differences can have on models created and that they cannot be appreciated in studies that have used a single finite element modelling software for simulation of lens shape change [25–27]. The findings of this study also have implications for intraocular lens (IOL) design. The effect of the zonule on the lens is important for the effective accommodating IOLs. Hence, even subtle differences in the deformation of the capsule modelled using different software can affect potential design and predicted performance of the IOL.

Finite element modelling of biological systems has increased in the last two decades and is an integral complement to experimental investigations. Modelling of the accommodative system has enabled studies of regions that are difficult to image and hence difficult to accurately measure experimentally. However, as with any investigative method, modelling has its limitations. The models and the results that they produce are dependent on input parameters. They are also influenced by the software packages.

The findings of this study show that even with identical input parameters, lens shape and zonular insertions, there will be differences in optical and biomechanical results depending on which Finite Element modelling software package is used. The stress ranges vary significantly with higher localised stresses seen in models created with one package than with another. Indeed, there were greater differences between models created with Ansys and Abaqus for the same zonular combination than between models with different zonular combinations created with one software package. Further refinement of software that would allow a combination of Ansys and Abaqus in a hybrid approach would increase opportunities for producing models that more closely mimic the biological tissue. It is notable that although accommodative capacity and its loss are age-related, individual variations in lens shape, size and refractive index need to be recognised [15]. Additionally, the two lenses used in this study were specifically chosen to demonstrate that results from a single lens model should not be treated as indicative of all lenses from the same age range.

### Supplementary Information

Below is the link to the electronic supplementary material.

Supplementary file1 (DOCX 527 kb)
