# Supplementary material for: The Effect of Lens Shape, Zonular Insertion and Finite Element Model on Simulated Shape Change of the Eye Lens
Source: Ann Biomed Eng. 2024 Mar 19;52(8):1982–90. doi: 10.1007/s10439-024-03491-3 (PMC11247046; doi:10.1007/s10439-024-03491-3)
Supplement: Supplementary file 1 — Supplementary file1 (DOCX 527 kb) [file 10439_2024_3491_MOESM1_ESM.docx]

**Supplementary material**


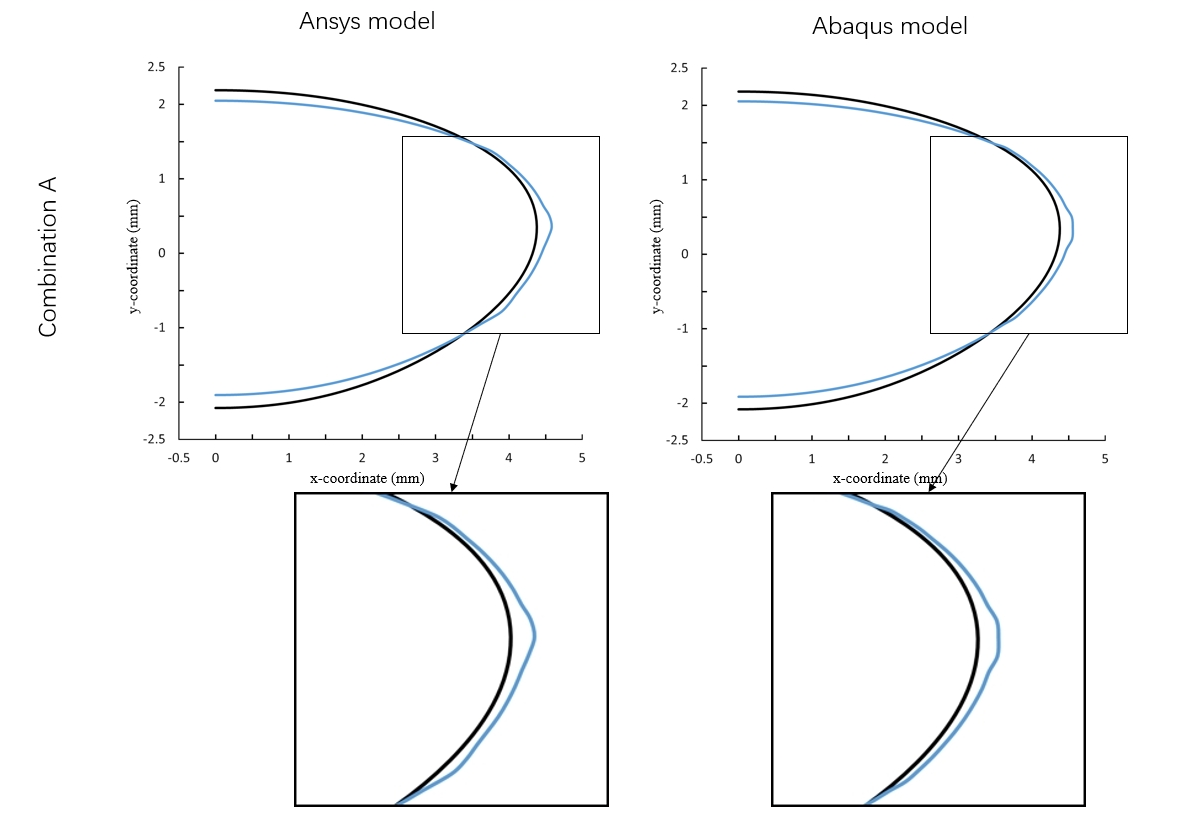


**Supplementary Figure S1** Comparison of lens external shapes before and after deformation in the asymmetric model under combination A.


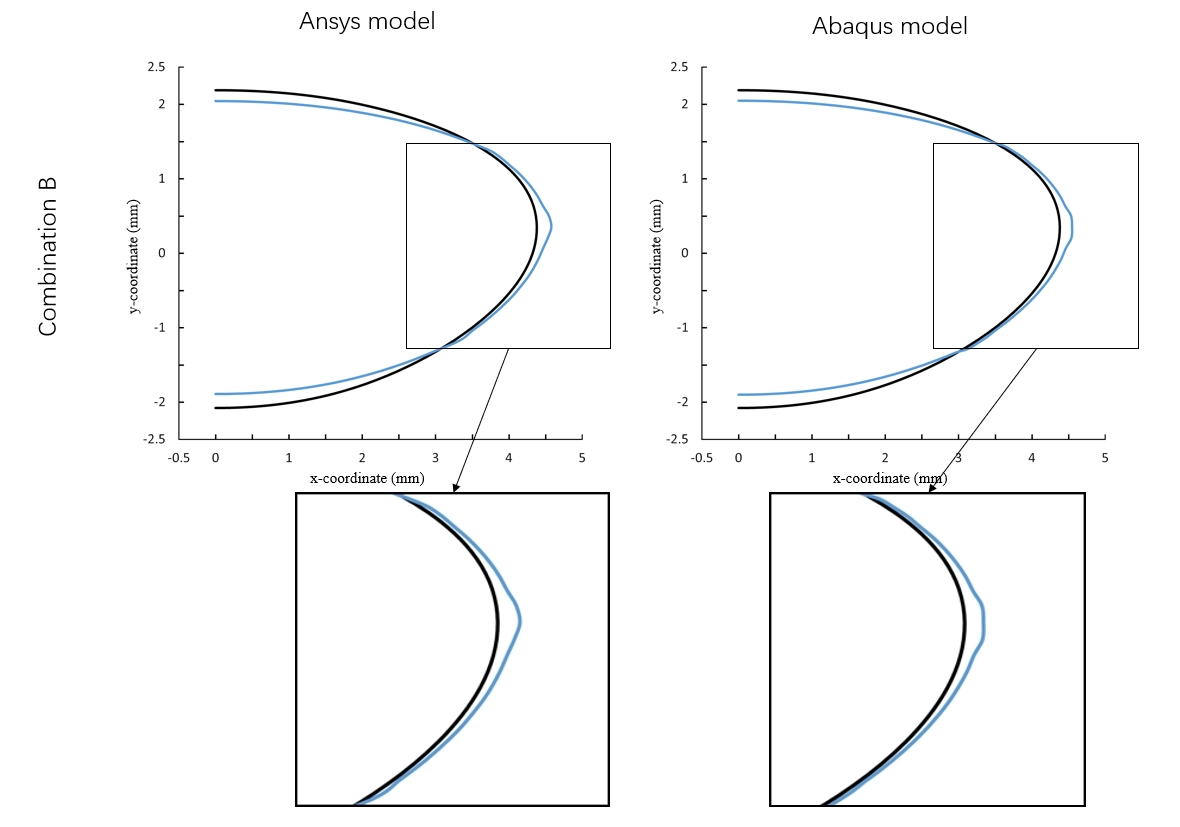


**Supplementary Figure S2** Comparison of lens external shapes before and after deformation in the asymmetric model under combination B.


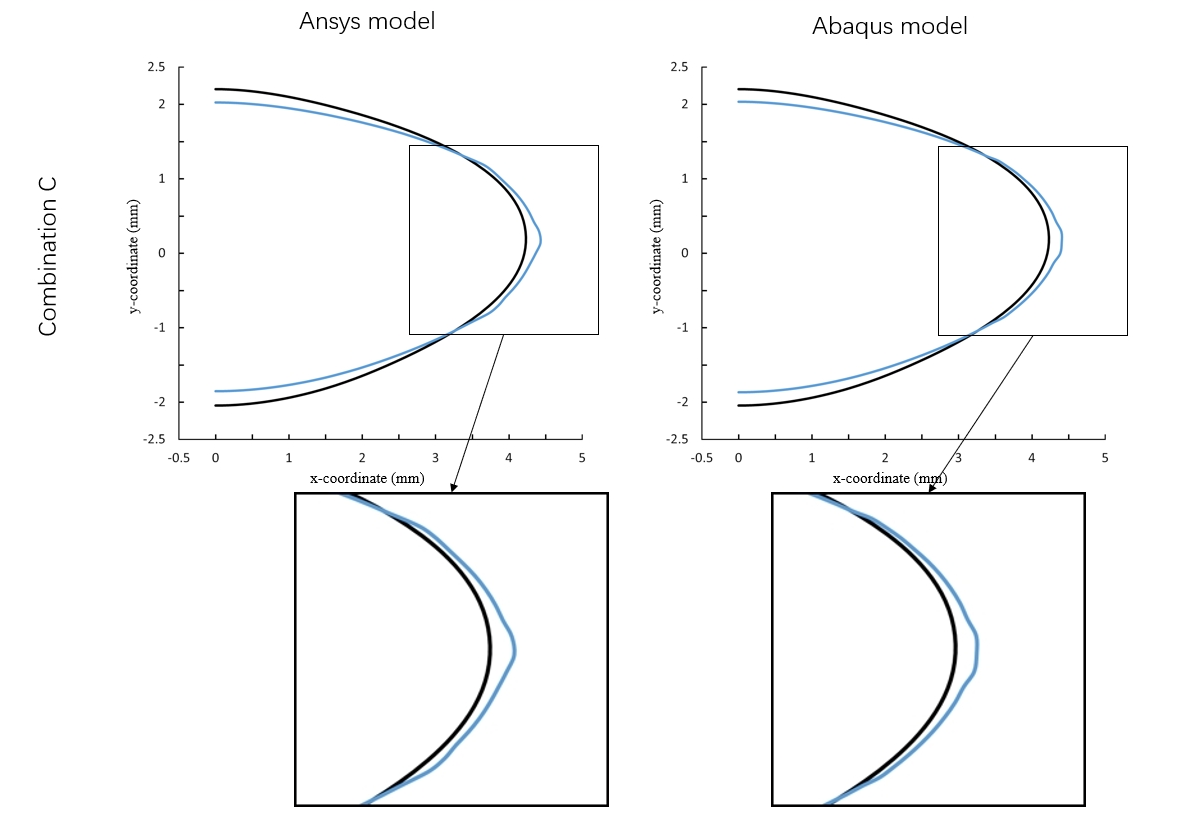


**Supplementary Figure S3** Comparison of lens external shapes before and after deformation in the symmetric model under combination C.


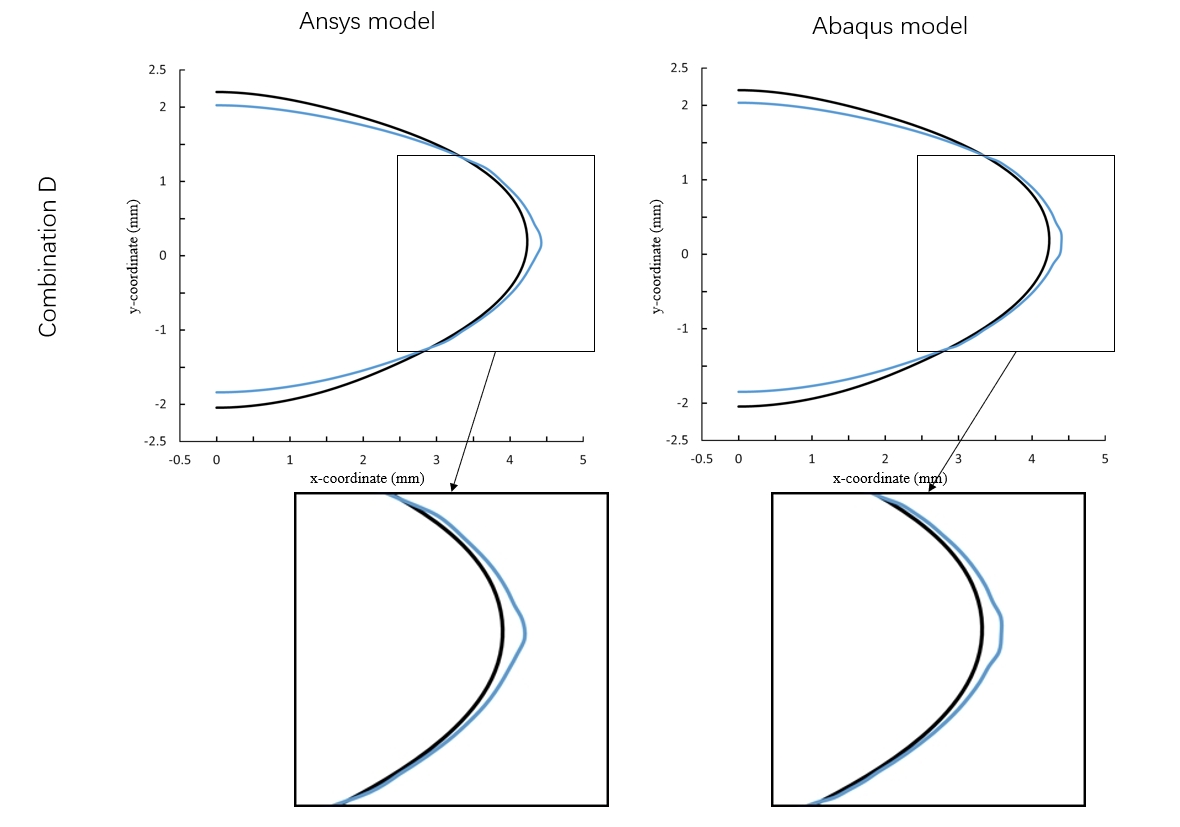


**Supplementary Figure S4** Comparison of lens external shapes before and after deformation in the symmetric model under combination D.
